# Challenges of Treatment-Resistant Bipolar Depression in the Elderly: A Case Study of Successful Modafinil Augmentation

**DOI:** 10.1192/bjo.2025.10748

**Published:** 2025-06-20

**Authors:** Arinze Mbanusi, Muhammad-Kazim Kanani

**Affiliations:** Tees, Esk and Wear Valleys Foundation NHS Trust, Durham, United Kingdom

## Abstract

**Aims:** Bipolar depression remains a major therapeutic challenge, particularly in elderly populations where treatment options are constrained by comorbidities and medication tolerability. Despite pharmacological advancements, many patients experience persistent depressive symptoms, even after multiple trials of mood stabilizers, antipsychotics, and antidepressants. Lithium, while effective, is associated with nephrotoxicity, limiting its use in the elderly. Modafinil, a wakefulness-promoting agent primarily used for narcolepsy, has emerged as a potential adjunctive treatment for treatment-resistant bipolar depression (TRBD). Its broad neurochemical effects, targeting multiple pathways, suggest promise in addressing refractory mood symptoms. However, its role in elderly patients remains underexplored.

**Methods:** We report the case of a 74-year-old Caucasian male with a long-standing history of bipolar disorder and multiple comorbidities, including hypertension, pulmonary fibrosis, cerebellar ataxia, and chronic kidney disease (CKD). His psychiatric symptoms began in 1995 with depression, progressing to a bipolar diagnosis after a manic episode in 1997. Initial treatment with lithium and venlafaxine stabilized his mood for several years. However, lithium was discontinued after nine years due to worsening renal function, leading to recurrent depressive episodes characterized by anhedonia, hypersomnia, and cognitive decline. Multiple pharmacological trials, including olanzapine, lamotrigine, fluoxetine, aripiprazole, quetiapine, and lurasidone, failed to achieve symptom remission. A retrial of lithium proved ineffective and was discontinued due to further renal deterioration. Given the lack of effective options, modafinil was introduced which improved his Montgomery–Åsberg Depression Rating Scale (MADRS) score from 29/60 to 6/60, reflecting enhanced mood, motivation, and wakefulness without adverse effects.

**Results:** TRBD in the elderly is complicated by age-related pharmacokinetic changes, comorbidities, and sensitivity to side effects. Standard mood stabilizers like lithium are often contraindicated due to nephrotoxicity, while atypical antipsychotics can introduce metabolic or cognitive risks. Modafinil’s unique neurochemical profile offers a novel approach to addressing treatment-resistant symptoms. While concerns regarding potential side effects, such as cardiovascular risks or mania induction exist, recent studies suggest modafinil is generally well-tolerated with minimal mood switch. This supports emerging evidence that modafinil may serve as an effective adjunctive therapy in TRBD, particularly when conventional treatments are limited.

**Conclusion:** This case highlights the successful use of modafinil as an adjunctive treatment in an elderly patient with TRBD. The patient’s significant improvement in depressive symptoms and overall functionality, without adverse effects, suggests that modafinil may be a viable option for elderly patients unresponsive to conventional therapies. However, further research, including randomized controlled trials, is needed to establish clear guidelines for its use.

